# *Lysobacter changpingensis* sp. nov., a novel species of the genus* Lysobacter* isolated from a rhizosphere soil of strawberry in China

**DOI:** 10.1007/s12223-023-01058-8

**Published:** 2023-06-02

**Authors:** Bang-Yan Niu, Dong-Jun Ren, Fang-Bo Zhang, Hong-Tu Zhu, Hai-Lei Wei, Ming-Chao Ma, Miao Gao

**Affiliations:** Institute of Agricultural Resources and Regional Planning, Chinese Academy of Agricultural Sciences/Key Laboratory of Microbial Resources Collection and Preservation, Ministry of Agriculture and Rural Affairs, Beijing, 100081 China

**Keywords:** *Lysobacter* sp., Rhizosphere microbiota, New species, Phenotypic

## Abstract

In the present work, we characterized in detail strain CM-3-T8^T^, which was isolated from the rhizosphere soil of strawberries in Beijing, China, in order to elucidate its taxonomic position. Cells of strain CM-3-T8^T^ were Gram-negative, non-spore-forming, aerobic, short rod. Growth occurred at 25–37 °C, pH 5.0–10.0, and in the presence of 0–8% (w/v) NaCl. Phylogenetic analysis based on 16S rRNA gene sequences revealed that strain CM-3-T8^T^ formed a stable clade with *Lysobacter soli* DCY21^T^ and *Lysobacter panacisoli* CJ29^T^, with the 16S rRNA gene sequence similarities of 98.91% and 98.50%. The average nucleotide identity and digital DNA-DNA hybridization values between strain SG-8^ T^ and the two reference type strains listed above were 76.3%, 79.6%, and 34.3%, 27%, respectively. The DNA G + C content was 68.4% (mol/mol). The major cellular fatty acids were comprised of C15:0 iso (36.15%), C17:0 iso (8.40%), and C11:0 iso 3OH (8.28%). The major quinone system was ubiquinone Q-8. The major polar lipids were phosphatidylethanolamine (PE), phosphatidylethanolamine (PME), diphosphatidylglycerol (DPG), and aminophospholipid (APL). On the basis of phenotypic, genotypic, and phylogenetic evidence, strain CM-3-T8^T^ (= ACCC 61714^ T^ = JCM 34576^ T^) represents a new species within the genus *Lysobacter*, for which the name *Lysobacter changpingensis* sp. nov. is proposed.

**Supplementary Information:**

The online version contains supplementary material available at 10.1007/s12223-023-01058-8.

## Introduction

The genus *Lysobacter*, in the class Gammaproteobacteria and family Xanthomonadaceae, was first described by Christensen and Cook (1978) and emended by Park et al. (2008). It is non-fruiting, Gram-stain-negative, aerobic, gliding nature of bacteria with high DNA G + C contents (61.7–70.7% mol/mol), contain ubiquinone Q-8 as the major respiratory quinone and the major polar lipids are diphosphatidylglycerol (DPG), phosphatidylglycerol (PG), and phosphatidylethanolamine (PE). At present, there are 70 species with a validly published and correct name in the genus *Lysobacter* recorded on LPSN (https://lpsn.dsmz.de/genus/Lysobacter; Feb 2023). *Lysobacter* strains are ubiquitously distributed in various environments, most of them were isolated from Antarctic coastal sediment (Liu et al. 2022), soil (Srinivasan et al. 2009; Coil et al. 2014; Gross et al. 2016; Zhang et al. 2019), plant rhizosphere (Xiao et al. 2019), feces (Lee et al. 2022), sludge (Ye et al. 2015), estuary (Sang et al. 2016), spongin (Choi et al. 2018), and freshwater (Siddiqi and Im 2016). In this paper, the authors report a novel bacterial strain CM-3-T8^T^, which was isolated from the rhizosphere soil of strawberry. Analysis of the phylogenetic and phenotypic characteristics confirmed that strain CM-3-T8^T^ represents a novel species within the genus *Lysobacter*, for which the name *Lysobacter changpingensis* sp. nov. is proposed.

## Materials and methods

### Isolation and ecology

Rhizosphere soil samples of strawberries were collected in the Changping district, Beijing, China (116° 20′ E, 40° 22′ N). For isolation of bacteria, the samples were suspended in sterile water and serially diluted to 10^−5^, 10^−6^, and 10^−7^, then 100 µL from each dilution was spread on TSA plates. The TSA medium contained (per liter): tryptone 15 g, soy peptone 5 g, sodium chloride 5 g, pH 7.0. After 3 days of incubation at 30 °C, a colony was subcultivated on TSA medium and named CM-3-T8^T^ to taxonomic characterization. The strain CM-3-T8^T^ was maintained on glycerol (w/v) and stored at −80 °C.

### 16S RNA phylogeny

Genomic DNA of CM-3-T8^T^ was extracted using a DNA extraction kit (Biotech) by following the manufacturer’s instructions. PCR amplification of the 16S rRNA gene was performed with the universal primers 27F (5′-GAGTTTGATCCTGGCTCAG-3′) and 1492R (5′-ACGGCTACCTTGTTACGACTT-3′) (Farris and Olson 2007). PCR conditions were an initial denaturation step at 94 °C for 2 min followed by 35 cycles of 95 °C for 30 s denaturing, 55 °C for 30 s annealing, and 72 °C for 30 s followed by a 10 min final extension at 72 °C. Purified PCR products were sequenced by the Sangon Biotech (Shanghai, China; http://www.sangon.com/). We used NCBI’s BLAST search (http://www.ncbi.nlm.nih.gov/blast) and the EzTaxone server (www. ezbiocloud.net) to identify phylogenetic neighbors and calculate pairwise sequence similarities. Then, phylogenetic trees were reconstructed using the software MEGA 7.0 and based on maximum-likelihood (Felsenstein 1981), neighbor-joining (Saitou and Nei 1987), and minimum-evolution (Rzhetsky and Nei 1992) models with bootstrap values under 1000 replications (Mikkel 2016). Distances were calculated according to Kimura’s two-parameter model (1980).

### Physiology and chemotaxonomy

For this part of the study, cells are cultured in TSA medium under aerobic conditions, and all data presented are the average of three replicates. Cell morphology and size was observed by a transmission electron microscope at 30 k magnification (Hitachi, Model H-7500, acceleration voltage 80 kV). Gram staining was performed by Beveridge (2001). The optimal pH and temperature for growth of strain CM-3-T8^T^ and the reference strains *Lysobacter soli* DCY21^T^ and *Lysobacter panacisoli* CJ29^T^ were determined by incubating the strains on TSA medium at different temperatures (4, 25, 30, 37, 42, and 45 °C), different pH levels (pH 4.0, 5.0, 6.0, 7.0, 8.0, 9.0, 10.0; acetate buffer was used for pH 4.0–7.0 and phosphate buffer was used for pH 7.0–10.0), and different NaCl levels with 0–10.0% (w/v) NaCl (1% increments). All the growth of the bacterium was determined by measuring the A_600_ (infinite M200PRO, TECAN) of the cultures after 5 days, except for the temperatures of 4 and 10 °C, which was assessed after 10 days. Enzyme activities were assayed using the API ZYM and API 20NE systems. Additional biochemical tests were determined using the API 50CH system and the Bio GN2 microplate according to the manufactures’ instructions.

For assaying the differences of fatty acid composition between the strain CM-3-T8^T^ and the most closely related species, strain CM-3-T8^T^, *Lysobacter soli* DCY21^T^, and *Lysobacter panacisoli* CJ29^T^ were used. The strains were cultured under aerobic conditions on TSA medium at 30 °C for 48 h. Fatty acid methyl esters were prepared and identified with the MIDI Sherlock Microbial Identification System (Sherlock version 6.1).

The polar lipids were extracted from 1 g freeze-dried cells using methanol/chloroform/saline extraction (2:1:0.8 ratio by vol.), as described by Kates (1972). Two-dimensional chromatography on a silica gel thin-layer chromatography (TLC) plate (10 × 10 cm) was used to separate and identify polar lipids, as described by Raj et al. (2013). Total polar lipids were detected by spraying with 10% ethanolic molybdophosphoric acid solution (Sigma-Aldrich) followed by heating at 150 °C for 10 min, and further characterized by spraying with ninhydrin, molybdenum blue (specific for phosphates), and Dragendorff’s reagent. The quinones were isolated according to the methods of Minnikin et al. (1984) and determined using HPLC (Kroppenstedt 1982).

### Genome features

The High Pure PCR Template Preparation kit (Roche) was employed for isolation of genomic DNA for whole-genome sequencing and DNA-DNA hybridization experiments. The genome of strain CM-3-T8^T^ was sequenced at Sistemas Genomicos (Valencia, Spain) using Illumina paired-end sequencing technology. The reads were trimmed using Trimmomatic 0.32 (Bolger et al. 2014). Genome assembly was performed using SPAdes 3.6.1 (Nurk et al. 2013). The average nucleotide identity blast (ANIb) values were calculated as described by Richter and RossellóMóra (2009) using JSpecies (version 1.2.1) and Lee et al. (2016). The G + C content of chromosomal DNA was calculated on the basis of its whole-genome sequence. The estimated DNA-DNA hybridization (dDDH) value was determined among these strains using the Genome-to Genome Distance Calculator (version 2.1) (Auch et al. 2010; Meier-Kolthoff et al. 2013).

## Results and discussion

### Molecular phylogenetic analysis

**Fig. 1:**
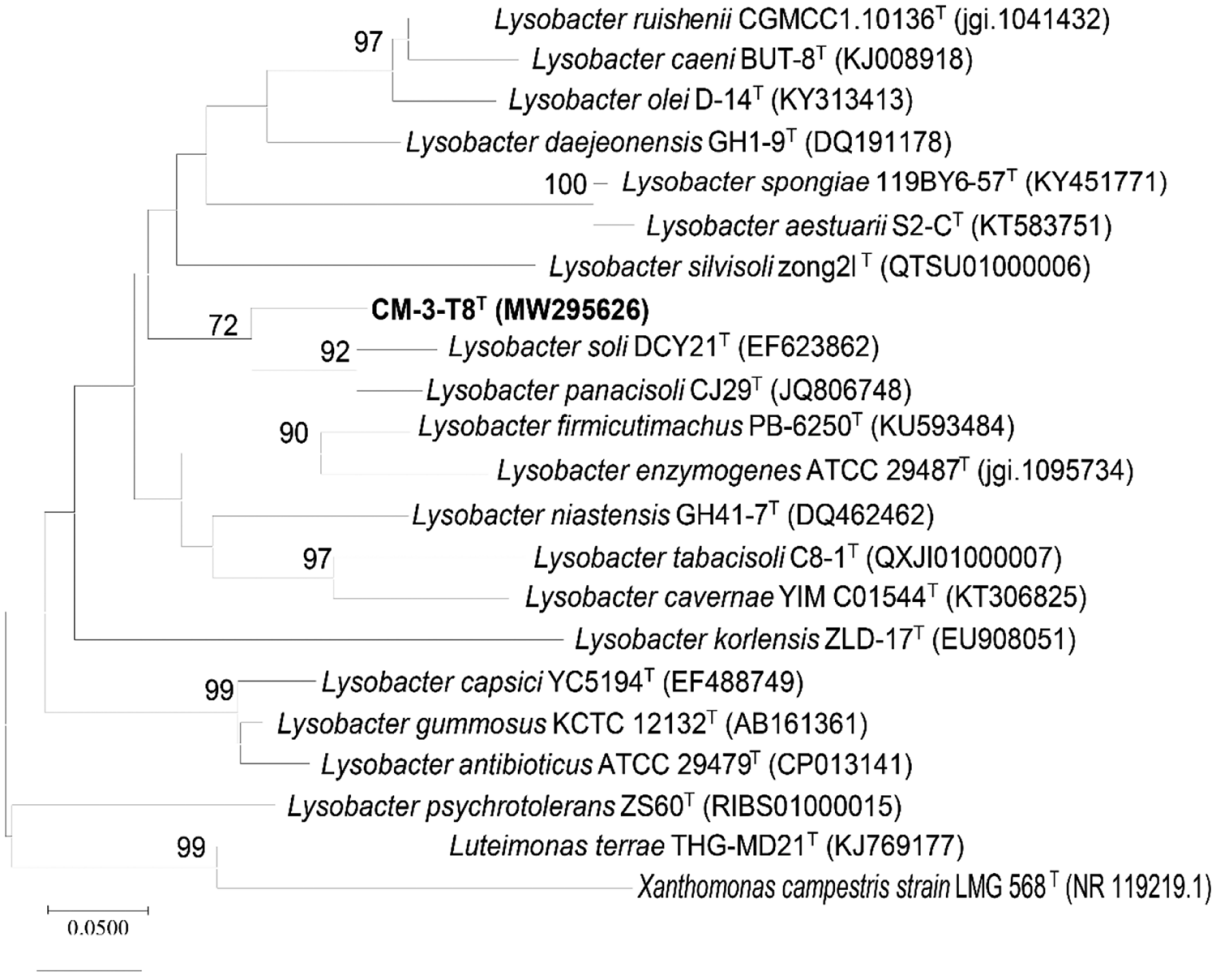
The maximum-likelihood (ML) tree based on partial 16S rRNA gene (1210 bp) sequence comparison showing the relationships between strain CM-3-T8^T^ and related strains of the family *Lysobacter*; bootstrap values > 50%, based on 1000 replications, are shown at branch points; bar corresponds to 0.005 substitutions per nucleotide position. *Xanthomonas campestris* strain LMG 568^ T^ was using as the outgroup

The 16S rRNA gene sequence (1408 bp) used NCBI’s BLAST search (http://www.ncbi.nlm.nih.gov/blast) and the EzTaxone server (www.ezbiocloud.net) to identify phylogenetic neighbors and calculate pairwise sequence similarities. *Lysobacter soli* DCY21^T^ and *Lysobacter panacisoli* CJ29^T^ exhibited the greatest similarity to the strain CM-3-T8^T^ (98.84% and 98.44% identities). The 16S rRNA gene sequence was deposited in the GenBank/EMBL/DDBJ database under accession number MW295626. The phylogenetic trees (Figs. 1, S1, S2) demonstrated that strain CM-3-T8^T^ belonged to the genus *Lysobacter* and formed a cluster with strains *Lysobacter soli* DCY21^T^ and *Lysobacter panacisoli* CJ29^T^ and other type strains were in different clades dispersedly in the maximum-likelihood, neighbor-joining, and minimum-evolution trees. In conclusion, *Lysobacter soli* DCY21^T^ and *Lysobacter panacisoli* CJ29^T^ were chosen as reference strains for further study.

### Physiology and chemotaxonomy analysis

**Fig. 2:**
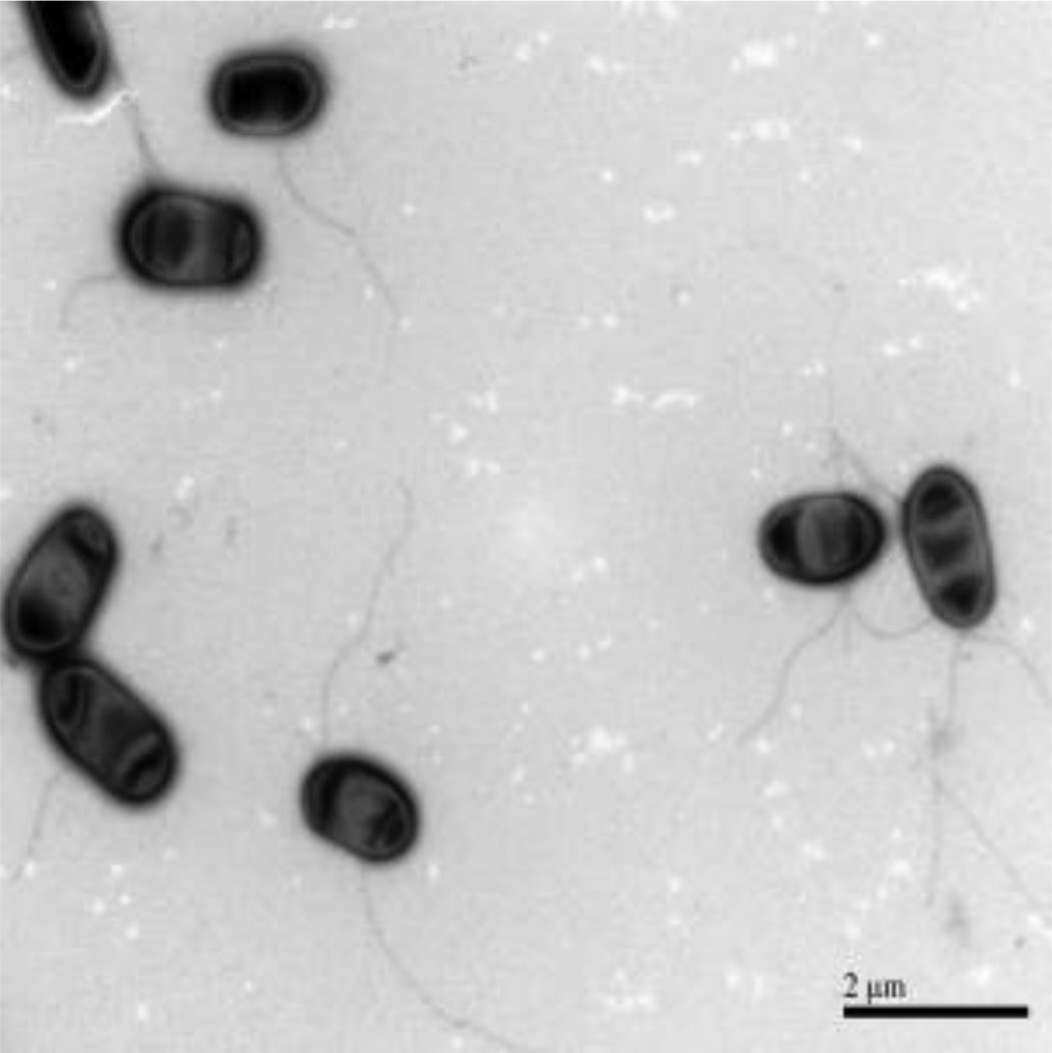
Scanning transmission electron microscopic of cells of strain CM-3-T8^T^; cells were cultured in TSA medium at 30 °C for 20 h; scale bar 2 μm

Cells of strain CM-3-T8^T^ were Gram-negative, non-spore-forming, aerobic, short rods (0.4–0.7 μm × 0.8–1.6 μm), commonly observed as single cells under the microscope (Fig. 2). Strain CM-3-T8^T^ can grow at 25–37 °C, pH 5.0–10.0, and in the presence of 0–8% (w/v) NaCl. The results of enzyme activities and other biochemical tests were listed in the species description (Table 1). Although strain CM-3-T8^T^ shared many phenotypic features with closely related taxa *Lysobacter soli* DCY21^T^ and *Lysobacter panacisoli* CJ29^T^, there were some differences among them. Strain CM-3-T8^T^ showed N-acetyl-D-glucosamine, N-acetyl-β-D-mannosamine, L-glutamic acid, and lithium chloride reactions are positive, but *L. panacisoli* CJ29^T^ and *L. soli* DCY21^T^ were negative for these characteristics. Whole cell fatty acid analysis revealed that the predominant fatty acids in strain CM-3-T8^T^ were C_15:0_ iso (36.15%), C_17:0_ iso (8.40%), and C_11:0_ iso 3OH (8.28%). These results were in line with other members of the genus *Lysobacter*. Nevertheless, there were several differences in the proportions of some fatty acids, such as more C_15:0_ iso and C_15:0_ anteiso (Table 2). The major polar lipids of strain CM-3-T8^T^ contain phosphatidylethanolamine (PE), phosphatidylethanolamine (PME), diphosphatidylglycerol (DPG), aminophospholipid (APL), small account of phosphatidylmonomethylethanolamine (PL), phosphatidylglycerol (PG), and unknownpolarlipids (Fig. S3). Q-8 was found to be the major quinone, in agreement with other members of the genus *Lysobacter* (Fig. S4).

**Table 1:** Differential phenotypic characteristics between strain CM-3-T8^T^ and closely related type strains in the genus *Lysobacter*

**Characteristics**^a^	**1**^b^	**2**^b^	**3**^b^
Morphology	Short rods	Rod	Rod
pH range for growth (optimum)	5–10 (7–9)	5–11 (7)	5–10.5 (7–7.5)
Temperature range for growth (°C) (optimum)	25–37 (30)	10–42 (30)	4–42 (30)
NaCl for growth (%, W/V) (optimum)	0–8	0–1 (1)	N
**BIOLOG**			
pH 6	+	+	W
N-Acetyl-D-glucosamine	+	−	−
N-Acetyl-β-D-mannosamine	+	−	−
1% NaCl	+	+	W
D-Galactose	+	+	W
L-Fucose	+	W	W
L-Rhamnose	W	−	−
1% sodium lactate	+	−	−
D-Fructose-6-PO_4_	−	−	W
L-Glutamic acid	+	−	−
Guanidine HCl	+	−	W
D-Galacturonic acid	+	+	−
D-Glucuronic acid	+	+	W
Lithium chloride	+	−	−
Tween 40	W	−	−
Sodium butyrate	−	−	+
**API ZYM**			
Cystinol arylamidase	+	+	W
Trypsin	W	W	−
Chymotrypsin	+	+	−
α-Glucosidase	+	+	−
β-Glucosidase	−	−	+
**API 20NE**			
4-Nitroso-β-D-methyl galactose	W	W	−
D-Glucose	+	W	+
Gluconate	W	W	−
**API 50CH**			
Galactose	W	−	W
Glucose	−	W	W
Geranyl	−	−	W
D-Lyxose	W	−	W
D-Fucose	+	−	W
5-Keto-gluconate	−	W	−

^a^( +)—positive, ( −)—negative, (W)—weakly positive; (N)—no data

^b^Strains: 1—CM-3-T8^T^ (this study); 2—*Lysobacter panacisoli* CJ29^T^ (this study); 3—*Lysobacter soli* DCY21^T^ (this study)

**Table 2:** Fatty acid compositions of strain CM-3-T8^T^ and closely related type strains in the genus *Lysobacter*

**Fatty acid**^a^	**1**^b^	**2**^b^	**3**^b^
**Saturated fatty acid**			
C_14:0_	0.32	0.42	0.35
C_16:0_	1.87	2.52	1.64
**Branched**			
C_11:0_ iso	6.04	7.66	6.01
C_13:0_ iso	0.22	0.45	0.20
C_14:0_ iso	0.55	0.60	0.46
C_15:1_ iso F	0.88	3.08	0.24
C_15:0_ iso	36.15	35.60	34.45
C_15:0_ anteiso	2.28	0.56	1.92
C_16:0_ iso	5.83	2.91	5.54
C_17:0_ iso	8.40	7.23	12.13
C_17:0_ anteiso	0.39	–	0.42
**Cyclopropane acids**			
C_17:0_ cyclo	0.20	0.12	–
**Hydroxy**			
C_11:0_ iso 3OH	8.28	9.48	7.34
Sum In Feature 3^c^	5.54	2.44	4.88
Sum In Feature 9^c^	20.10	25.64	22.79

^a^Values are percentages of total fatty acids; (–)—not detected

^b^Strains: 1—CM-3-T8^T^ (this study); 2—*Lysobacter panacisoli* CJ29^T^ (this study); 3—*Lysobacter soli* DCY21^T^ (this study)

^c^Sum In Feature 3 comprises C_16:1_ ω7c/C_16:1_ w6c or C_16:1_ ω6c/C_16:1_ ω7c, Sum In Feature 9 comprises C_16:1_ 10-methyl or C_17:1_ iso ω9c

### Genome features

**Fig. 3:**
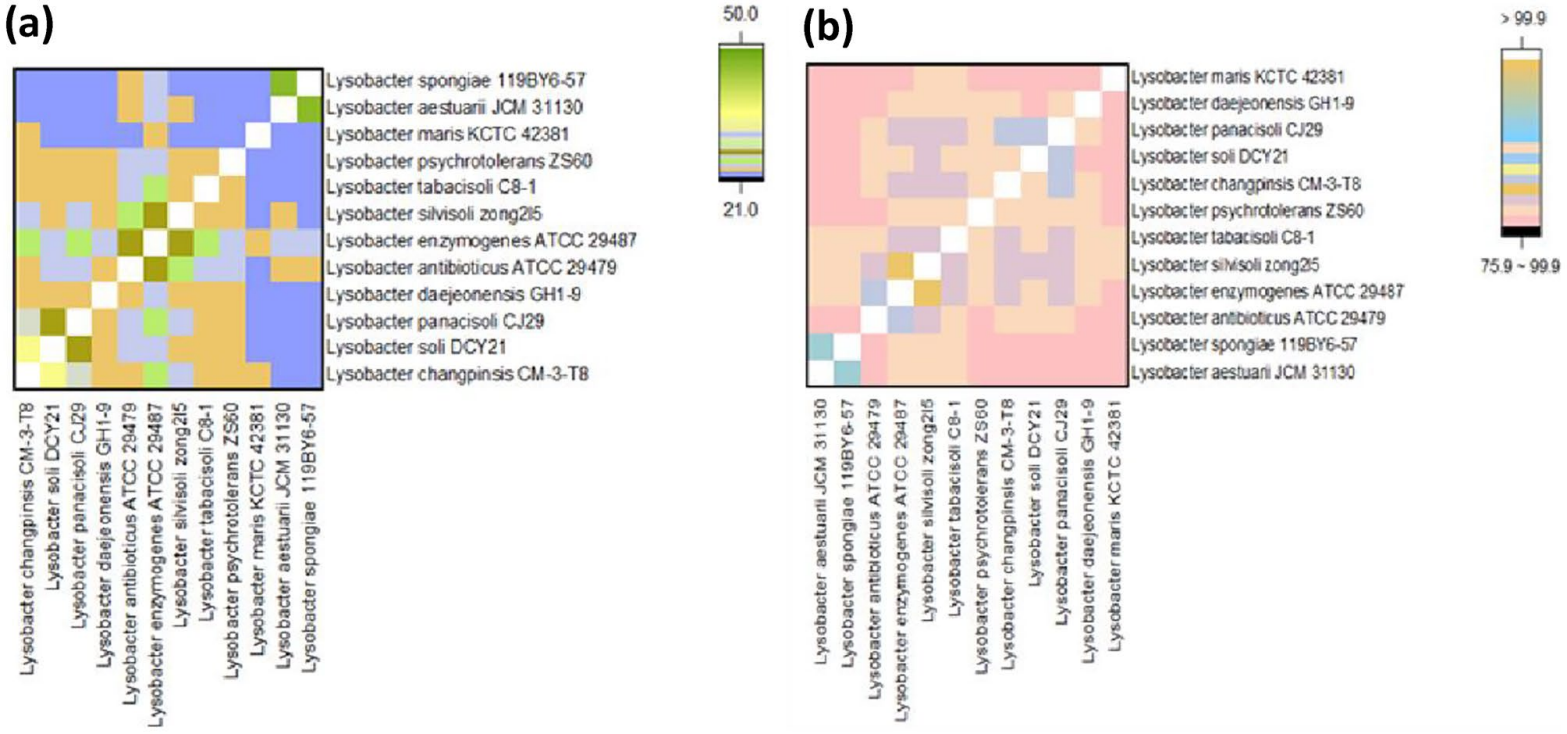
Analysis of *Lysobacter* genomes. **a** The DNA-DNA hybridization (dDDH) values between CM-3-T8^T^ and the selected reference strains; **b** overall orthologous average nucleotide identity (ANI) among pairwise *Lysobacter* genomes. Values in heatmap indicate the similarity percentage

**Fig. 4:**
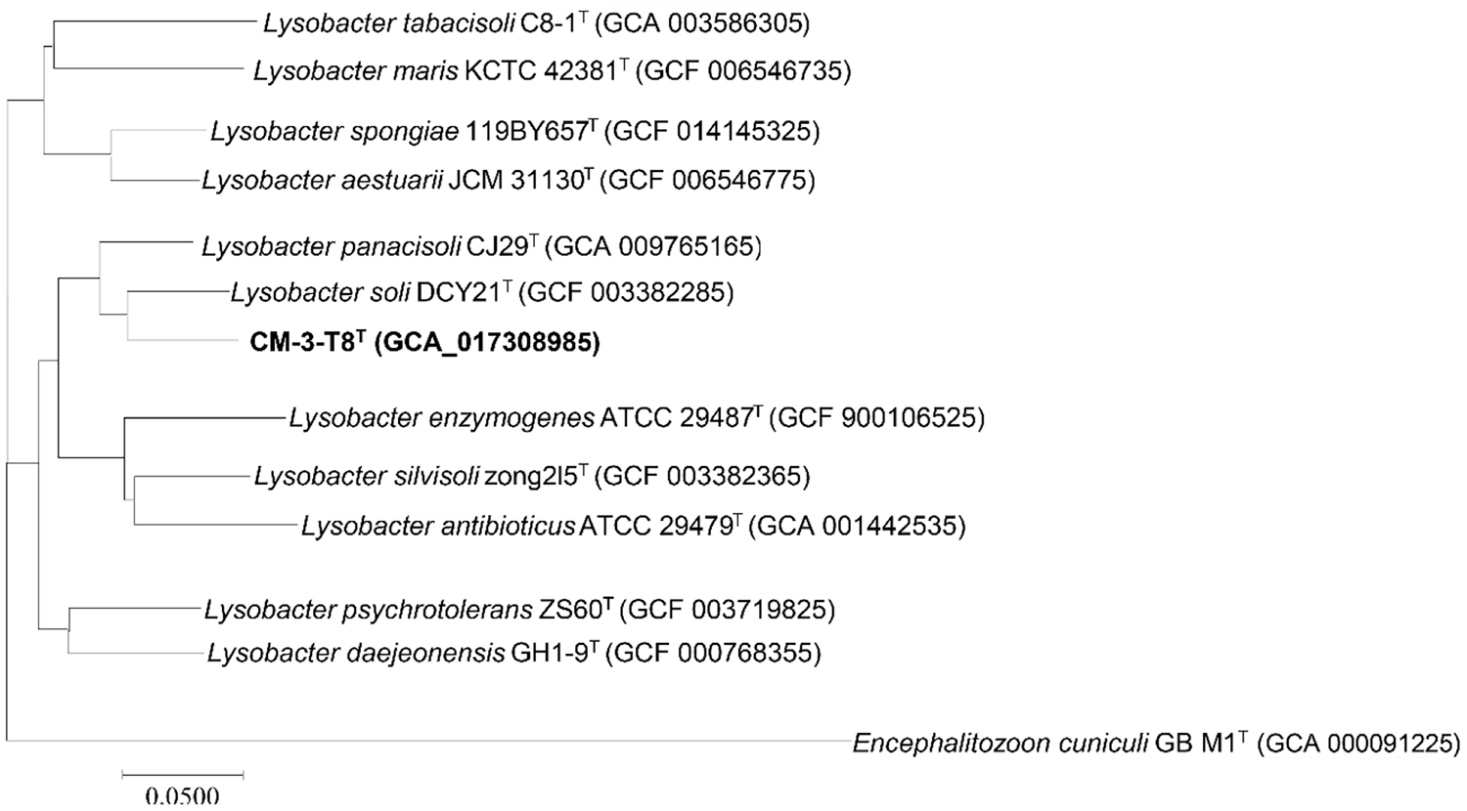
Phylogenomic tree generated with Genome-to-Genome Distance Calculator (GGDC); the numbers at the nodes indicate the gene support index; bar corresponds to 0.02 substitutions per position. Strain *Encephalitozoon cuniculi* GB M1^T^ was using as outgroup

The DNA G + C content of strain was estimated at 68.4% (mol/mol) according to the draft genome of strain CM-3-T8^T^, which is in the range of the genus *Lysobacter* 66.8–72.2% (mol/mol). The estimated DNA-DNA hybridization (dDDH) values for strain CM-3-T8^T^ with strains *Lysobacter soli* DCY21^T^ and *Lysobacter panacisoli* CJ29^T^ were 34.3% and 27%, respectively. The average nucleotide identity (ANI) values of CM-3-T8^T^ with the *Lysobacter soli* DCY21^T^ and *Lysobacter panacisoli* CJ29^T^ are between 76.3 and 79.6% (Fig. 3). The average nucleotide identity and in silico estimated DNA-DNA reassociation values among strain CM-3-T8^T^, strains *Lysobacter soli* DCY21^T^ and *Lysobacter panacisoli* CJ29^T^ were in all cases below the respective threshold for species differentiation (95–96% for ANI, 70% for dDDH) (Lee et al. 2016), suggesting that strains be proposed as a novel species of the genus *Lysobacter*. The whole-genome phylogenetic tree was constructed with other eleven publicly available *Lysobacter* species genomes (Fig. 4), showing the close phylogenetic relationship between closely related type strains *Lysobacter panacisoli* CJ29^T^, *Lysobacter soli* DCY21^T^ and strain CM-3-T8^T^, suggesting that strain CM-3-T8^T^ was affiliated to the genus *Lysobacter*. The genome of strain CM-3-T8^T^ was sequenced and compared to two reference genomes of *Lysobacter* species (Table 3). These genomic features can distinguish strain CM-3-T8^T^ from its closely related strains.

**Table 3:** Feature’s comparison of three *Lysobacter* sp. genomes

**Genomics feature**	***Lysobacter changpingensis* CM-3-T8**^T^	***Lysobacter panacisoli* CJ29**^**T**^	***Lysobacter soli* DCY21**^**T**^
Genome size (bp)	4,059,994	3,879,713	3,953,742
G + C content (% mol/mol)	68.24	67.5	67.65
Contigs	9	3	27
Contig N50	1,171,793	2,610,236	285,382
Component sequences (WGS or clone)	9	2	27
Protein	3635	3585	13,670
Gene	3635	3626	3741

*Lysobacter* spp. are widely distributed and exhibit remarkable bactericidal activity against a wide range of phytopathogenic fungi, bacteria, and nematodes. The vast majority of *Lysobacter* spp. adapt to the soil environment; among the 70 species of the genus, 56 species were isolated from soil. These soil types include greenhouse soil with relatively high humidity (Weon et al. 2006), dry soil with strong ultraviolet radiation (Zhang et al. 2011), saline-alkali soil (Xu et al. 2020), oil-contaminated soil (Chaudhary et al. 2017), and alpine forest soil (Margesin et al. 2018), indicating that *Lysobacter* bacteria have a wide range of adaptability in soil. The strain CM-3-T8^T^ in this study was also isolated from soil. Unlike other published strains of the genus *Lysobacter*, strain CM-3-T8^T^ can utilize multiple carbon sources, such as N-acetyl-D-glucosamine, N-acetyl-β-D-mannosamine, 1% sodium lactate, L-fucose, L-glutamic acid, guanidine HCl, lithium chloride, and D-fucose, which contributes to its distribution. N-acetyl-D-glucosamine is one of the monomers of chitin, glycosaminoglycans, and glycoproteins and plays an important role in the formation of microbial cell walls (Mobley et al. 1982). Strain CM-3-T8^T^ exhibits the ability to oxidize N-acetyl-D-glucosamine, which may contribute to its antibacterial activity. Many reported *Lysobacter* spp. have demonstrated significant antagonistic effects against a variety of pathogens. In our future work, we will study and evaluate the biocontrol potential and mechanism of strain CM-3-T8^T^. We will assess its biocontrol potential under different environmental conditions, including its ability to inhibit various plant pathogens and promote crop growth. Our studies will help to uncover the biocontrol potential and mechanism of CM-3-T8^T^, providing a theoretical basis and technical support for its application in agricultural production.

## Conclusion

In conclusion, the characteristics of the novel species are consistent with the description of the genus *Lysobacter* according to morphological, biochemical, and chemotaxonomic properties, but there are several differences between CM-3-T8^T^ and other published members of the genus *Lysobacter*. Phylogenetic and chemotaxonomic analyses demonstrate that strain CM-3-T8^T^ represents a novel species within the genus *Lysobacter*. The genome sequence of strain CM-3-T8^T^ was deposited in the GenBank/EMBL/DDBJ database under accession number GCA_017308985.

## Description of *Lysobacter changpingensis* sp. nov

### ***Lysobacter changpingensis***

(chang.ping.en’sis. N.L. masc. adj. changping of a district in Beijing of China, where the type strain was isolated).

Cells are Gram-negative, non-spore-forming, aerobic, short rods, commonly observed as single cells under the microscope. *Lysobacter changpingensis* can grow at 25–37 °C (optimum, 30 °C) and at pH 5.0–10.0 (optimum, pH 7.0–9.0). The salt tolerance range for growth is 0–8% (w/v) NaCl. The major cellular fatty acids are comprised of C_15:0_ iso (36.15%), C_17:0_ iso (8.40%), and C_11:0_ iso 3OH (8.28%). Reactions are positive for N-acetyl-D-glucosamine, N-acetyl-β-D-mannosamine, 1% NaCl, D-galactose, L-fucose, 1% sodium lactate, L-glutamic acid, guanidine HCl, D-galacturonic acid, D-glucuronic acid, and lithium chloride in BIOLOG strip; cystinol arylamidase, chymotrypsin, and α-glucosidase in API ZYM strip; D-glucose in API 20NE strip; and D-fucose in API 50CH strip. Resistant to D-fructose-6-PO_4_, sodium butyrate, β-glucosidase, glucose, geranyl, and 5-keto-gluconate, while weakly sensitive to L-rhamnose, tween 40, trypsin, 4-nitroso-β-D-methyl galactose, gluconate, galactose, and D-lyxose. Strain CM-3-T8^T^ mainly contains phosphatidylethanolamine (PE), phosphatidylethanolamine (PME), diphosphatidylglycerol (DPG), aminophospholipid (APL), small account of phosphatidylmonomethylethanolamine (PL), unknownpolarlipids (L), and phosphatidylglycerol (PG). The major quinone system is ubiquinone Q-8.

The type strain of *Lysobacter changpingensis* is CM-3-T8^T^ (= ACCC 61731^ T^ = JCM 33722^ T^), isolated from the rhizosphere of strawberry in Changping district, Beijing, China. The DNA G + C content is 68.24% (mol/mol). The 16S rRNA gene sequence is deposited in the GenBank/EMBL/DDBJ database under accession number MW295626. The genome sequence is deposited in the GenBank/EMBL/DDBJ database under accession number GCA_017308985.

### Supplementary Information

Below is the link to the electronic supplementary material.

Supplementary file1 (DOC 951 KB)

## Data Availability

All data generated during this study are publicly available from the GenBank database at http://www.ncbi.nlm.nih.gov/blast and http://www.ezbiocloud.net.
