# Supplementary material for: Lysobacter changpingensis sp. nov., a novel species of the genus Lysobacter isolated from a rhizosphere soil of strawberry in China
Source: Folia Microbiol (Praha). 2023 Jun 2;68(6):991–8. doi: 10.1007/s12223-023-01058-8 (PMC10689546; doi:10.1007/s12223-023-01058-8)
Supplement: Supplementary file 1 — Supplementary file1 (DOC 951 KB) [file 12223_2023_1058_MOESM1_ESM.doc]

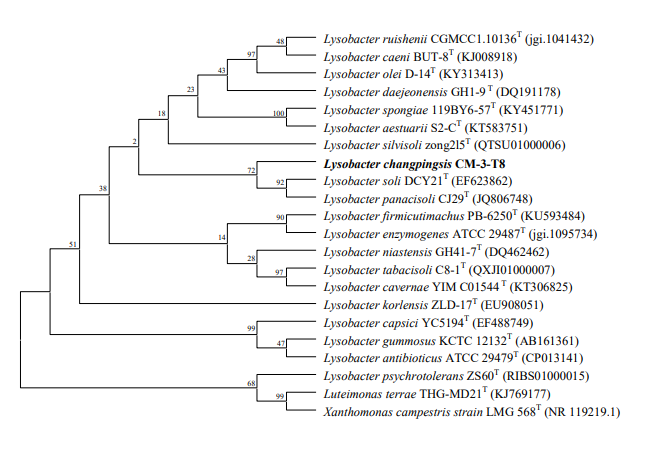


**Figure S1.** Neighbour-joining tree analysis between strain CM-3-T8 and closely related type strains in the genus *Lysobacter.* Bootstrap values >50%, based on 1000 replications, are shown at branch points. Bar, 0.01 substitutions per nucleotide position. *Xanthomonas campestris strain* LMG 568 ^T^ was using as the outgroup.


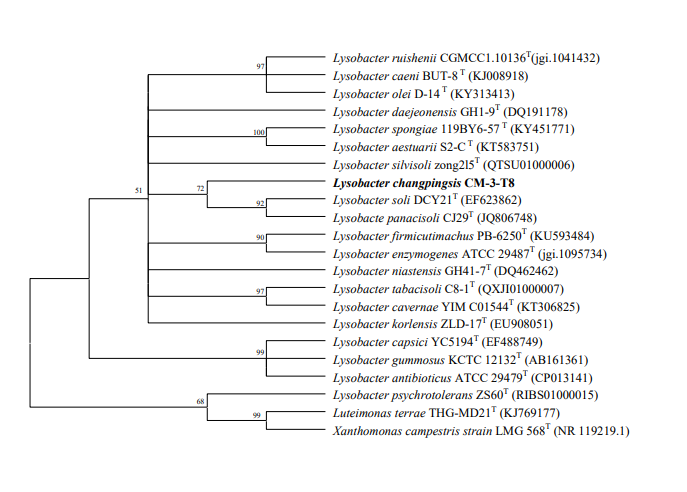


**Figure S2.** Minimum-evolution tree analysis between strain CM-3-T8 and closely related type strains in the genus *Lysobacter.* Bootstrap values >50%, based on 1000 replications, are shown at branch points. *Xanthomonas campestris strain* LMG 568 ^T^ was using as the outgroup.

a b c


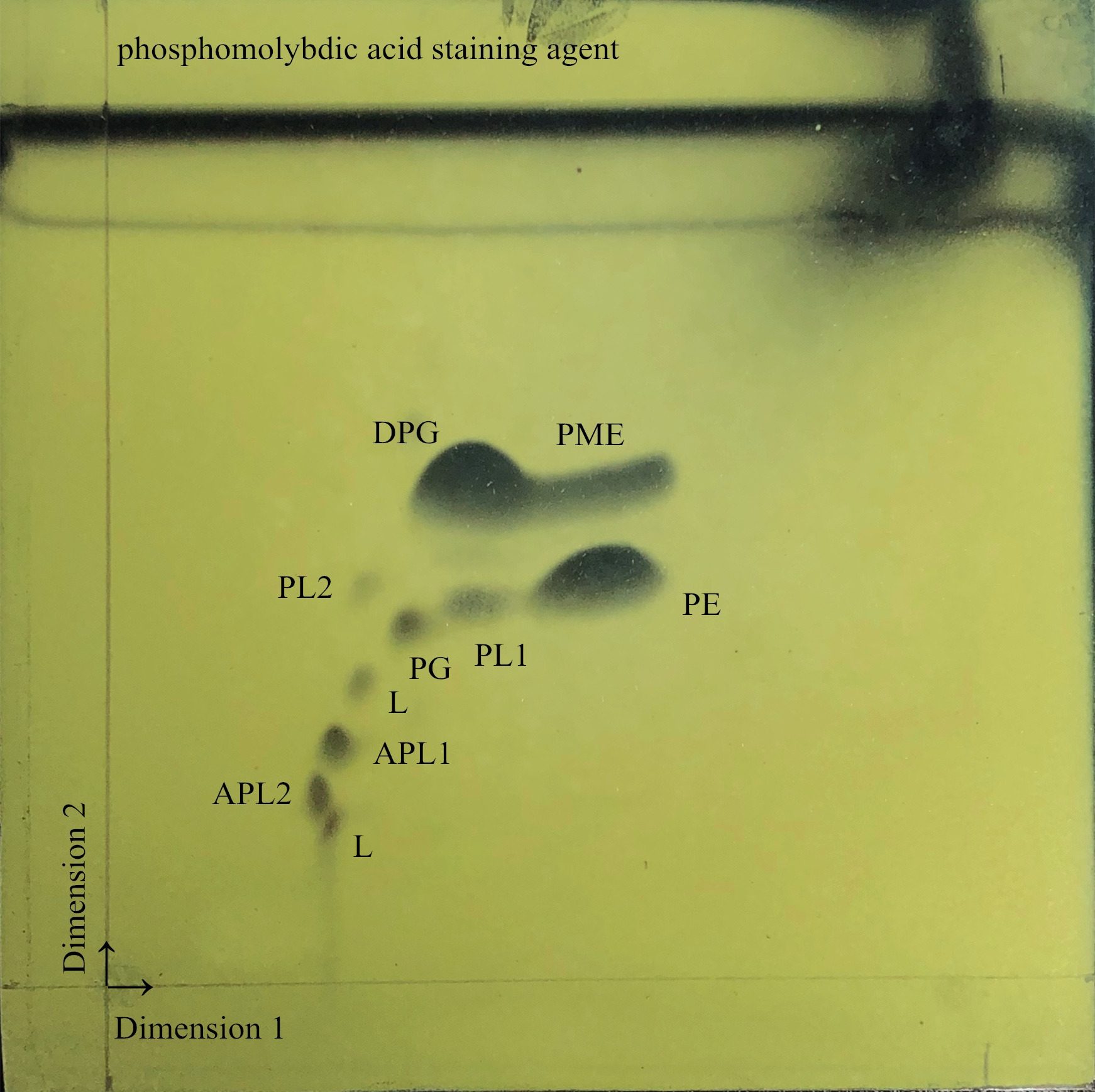

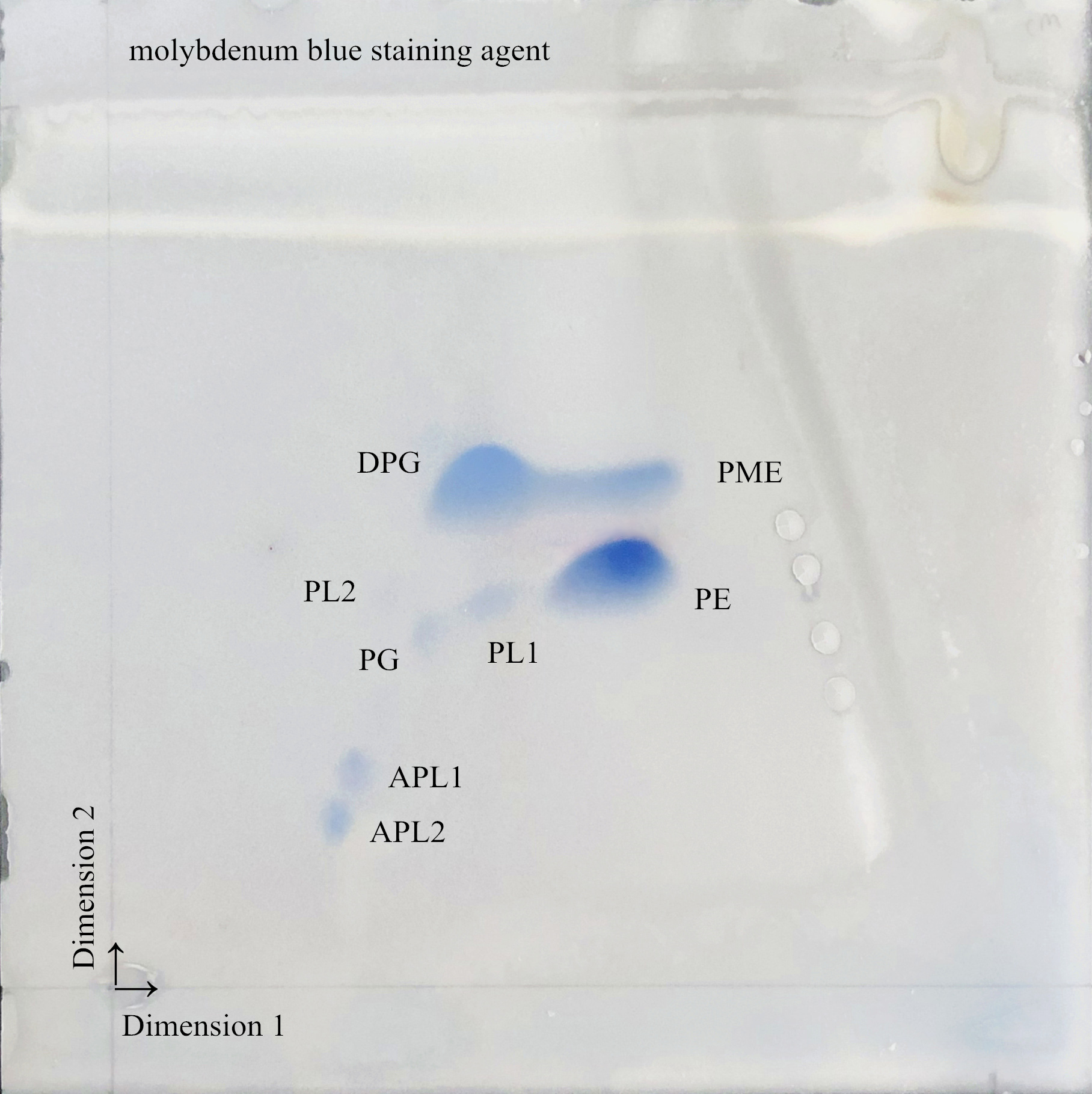

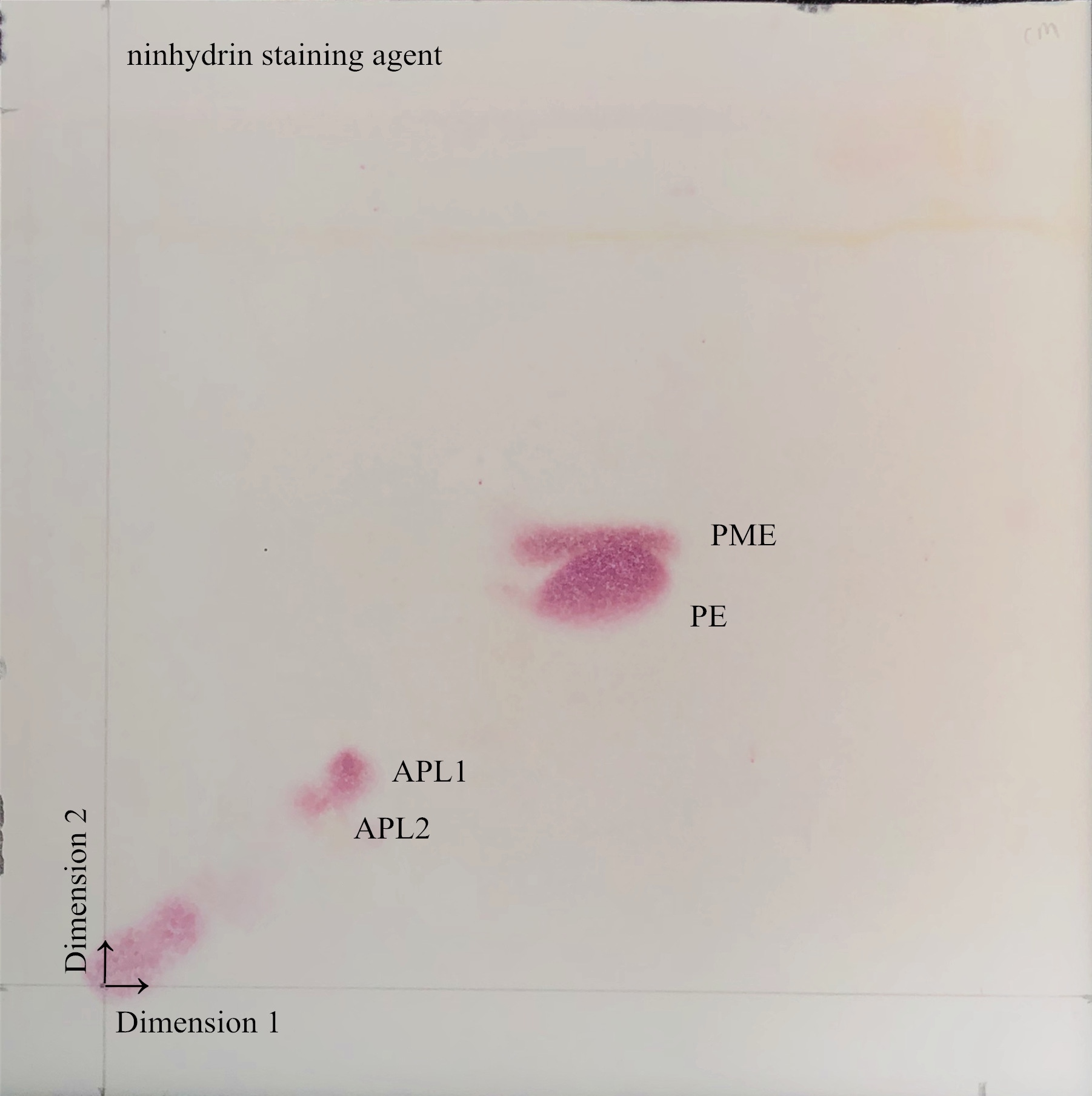


**Figure S3.** The polar lipids analysis of strain CM-3-T8. PE, phosphatidylethanolamine; PME, phosphatidymethylethanolamine; PG, phosphatidylglycerol; APL, aminophospholipid; DPG, diphosphatidylglycerol; PL, unidentified phosphoglycolipid. (a) molybdophosphoric acid spray reagent and heating at 150℃ for 10 min, (b) molybdenum blue spray reagent, and (c) ninhydrin spray reagent and heating at 100℃ for 10 min. No spots were detected by α-naphthhol spray reagent and heating at 100℃ for 5 min. Chloroform/methanol/water (65:25:4, by vol.) was used in the first direction.


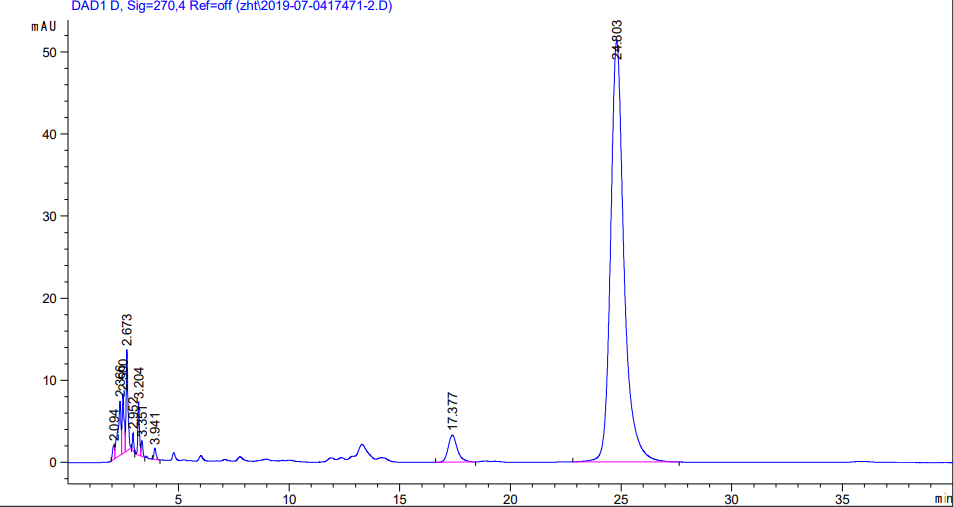


**Fig S4.** Respiratory quinone type of CM-3-T8.
